# Rapid prototyping compliant arterial phantoms for *in*-*vitro* studies and device testing

**DOI:** 10.1186/1532-429X-15-2

**Published:** 2013-01-16

**Authors:** Giovanni Biglino, Peter Verschueren, Raf Zegels, Andrew M Taylor, Silvia Schievano

**Affiliations:** 1 Centre for Cardiovascular Imaging, UCL Institute of Cardiovascular Science & Great Ormond Street Hospital for Children, NHS Trust, London, UK; 2 Materialise NV, Biomedical Engineering Business Unit, Leuven, Belgium

**Keywords:** Rapid prototyping, PolyJet, Phantoms, Compliance, TangoPlus FullCure®

## Abstract

**Background:**

Compliant vascular phantoms are desirable for *in*-*vitro* patient-specific experiments and device testing. TangoPlus FullCure 930® is a commercially available rubber-like material that can be used for PolyJet rapid prototyping. This work aims to gather preliminary data on the distensibility of this material, in order to assess the feasibility of its use in the context of experimental cardiovascular modelling.

**Methods:**

The descending aorta anatomy of a volunteer was modelled in 3D from cardiovascular magnetic resonance (CMR) images and rapid prototyped using TangoPlus. The model was printed with a range of increasing wall thicknesses (0.6, 0.7, 0.8, 1.0 and 1.5 mm), keeping the lumen of the vessel constant. Models were also printed in both vertical and horizontal orientations, thus resulting in a total of ten specimens. Compliance tests were performed by monitoring pressure variations while gradually increasing and decreasing internal volume. Knowledge of distensibility was thus derived and then implemented with CMR data to test two applications. Firstly, a patient-specific compliant model of hypoplastic aorta suitable for connection in a mock circulatory loop for *in*-*vitro* tests was manufactured. Secondly, the right ventricular outflow tract (RVOT) of a patient necessitating pulmonary valve replacement was printed in order to physically test device insertion and assess patient’s suitability for percutaneous pulmonary valve intervention.

**Results:**

The distensibility of the material was identified in a range from 6.5 × 10^-3^ mmHg^-1^ for the 0.6 mm case, to 3.0 × 10^-3^ mmHg^-1^ for the 1.5 mm case. The models printed in the vertical orientation were always more compliant than their horizontal counterpart. Rapid prototyping of a compliant hypoplastic aorta and of a RVOT anatomical model were both feasible. Device insertion in the RVOT model was successful.

**Conclusion:**

Values of distensibility, compared with literature data, show that TangoPlus is suitable for manufacturing arterial phantoms, with the added benefit of being compatible with PolyJet printing, thus guaranteeing representative anatomical finishing, and quick and inexpensive fabrication. The appealing possibility of printing models of non-uniform wall thickness, resembling more closely certain physiological scenarios, can also be explored. However, this material appears to be too stiff for modelling the more compliant systemic venous system.

## Background

Mock circulatory systems are a valuable tool for hydrodynamic studies and *invitro* device testing, whose complexity varies according to the purpose of the study [1]. Data gathered in a mock system can also serve as validation for computational studies [2]. These experimental setups can include anatomical phantoms attached to resistive and compliant elements according to a Windkessel type model [1], and in this setting phantoms can be either an idealised test section [3,4] or patient-specific models [5-7]. Traditionally, rigid models have been manufactured using resins [7,8] or glass [9]. While these are useful for visualisation studies, such as particle image velocimetry (PIV), they do not replicate the compliant nature of the vasculature and the associated Windkessel effect. Manufacturing flexible models, nonetheless, can be challenging, and methods such as dipping [3,10] or dripping [11] can be cumbersome, often with inadequate results. Different materials have been used for flexible vascular models, including silicone [2,11,12], polyurethane [13] and latex [14]. Recently, the dip-spin coating technique produced satisfactory results in terms of geometry, employing a stereolithography-generated mould dipped in silicone and dried during biaxial rotation, and models of bypass graft, aortic arch and abdominal aorta have been presented [15]. However, the distensibility of these models was not quantified and they had uniform wall thickness. Another interesting recent example is the use of an inner and outer stereo-lithographic mould with clear silicone rubber poured in the cast, resulting in a realistic aortic arch aneurysm model for endovascular prosthesis assessment [16]. This model also has uniform thickness, it lacks quantitative distensibility information and the process is admittedly not suitable for small vessels (i.e. paediatric applications).

In the search for realistic phantoms for vascular studies, rapid prototyping PolyJet technique nowadays allows for printing compliant models. PolyJet involves jetting state of the art photopolymer materials layer by layer, the layers being ultra-thin (16 μm) [17]. The advantages associated with PolyJet are the geometrical accuracy and finishing of the model, the speed of the process and its relatively low cost. A rubber-like, commercially available compound, namely TangoPlus FullCure® (Objet ltd, Rehovot, Israel), is compatible with PolyJet machines and is thus investigated here as a potential material for manufacturing compliant vascular models. Examples of TangoPlus being employed for manufacturing compliant anatomical phantoms are few and include a renal application [18] and simulation of cerebral aneurysm clipping [19].

To our knowledge, quantification of the material’s distensibility and probing its use in cardiovascular applications are currently lacking. This study thus aims to obtain preliminary distensibility data from cardiovascular magnetic resonance (CMR), thus using patient-specific information, and to assess the feasibility of this technique within a physiological range, presenting two examples of vascular anatomies, one adult and one paediatric.

## Methods

### Quantification of distensibility in a simple anatomical model

**Figure 1:**
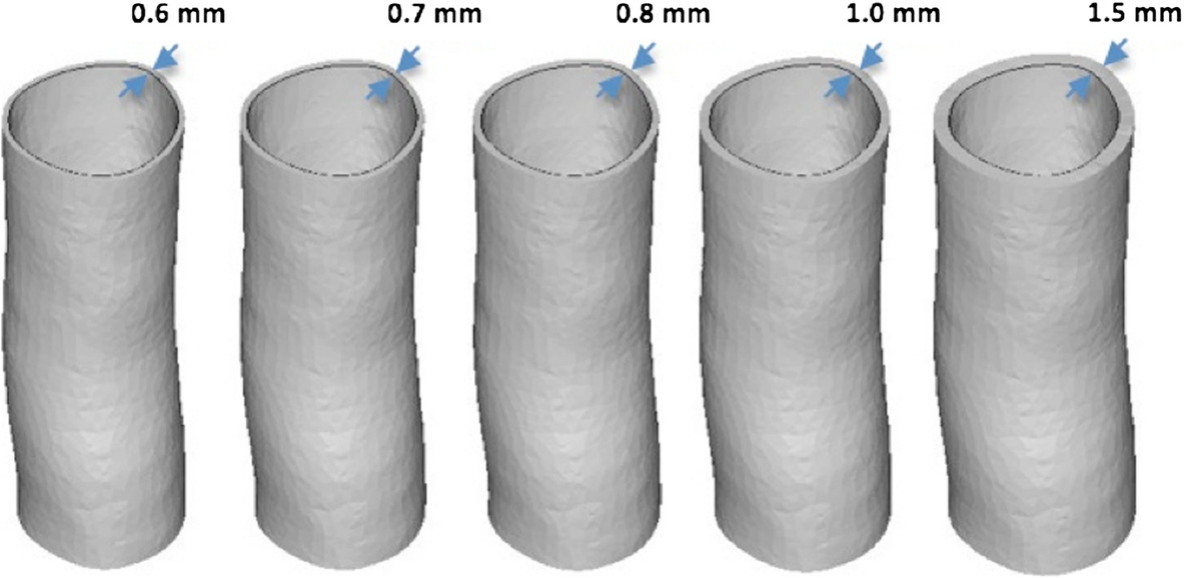
**Aortic models. **Reconstructed tract of descending aorta, extruded five times with increasing wall thickness (0.6, 0.7, 0.8, 1.0 and 1.5 mm) keeping the internal diameter constant (15.5 mm).

A 50 mm tract of descending aorta of a 29-year-old volunteer was imaged with CMR. The anatomy was reconstructed in 3D from the whole heart sequence using commercial software (Mimics®, Materialise NV, Leuven, Belgium) following a procedure described elsewhere [20]. Use of imaging data for research purposes is approved by the Local Research Committee. The volunteer gave informed consent for the CMR study and the use of data for research purposes. The reconstructed geometry represented the lumen of the vessel and it was extruded (3-matic®, Materialise NV) with increasingly thicker walls (0.6, 0.7, 0.8, 1.0 and 1.5 mm), as shown in Figure 1. These thicknesses were selected arbitrarily, with 0.6 mm indicated by the manufacturer as a limit below which the structure would collapse during the printing process. All models had the same lumen (15.5 mm internal diameter). The five specimens were printed using a PolyJet machine (Eden 500V, Objet ltd) and TangoPlus FullCure® material. Each model was printed in two orientations – defined either “vertical” or “horizontal”, according to the direction of printing – and a set of ten models was finally generated.

A compliance test was carried out for each descending aorta model. Each was filled with water and a closed volume was obtained by blocking the model’s extremities with suitable lids. Internal pressure (P) was continuously monitored using a high-fidelity fiber-optic sensor (Preclin 420, Samba Sensors, Västra Frölunda, Sweden) inserted into the model from one of the extremities, while the internal volume (V) was varied by an amount ΔV = 2.5 ml in steps (dV) of 0.5 ml. Data were recorded during loading and unloading at 250 Hz (AcqKnowledge, Biopac systems Inc, Goleta, CA, USA). The experiments were carried out at room temperature. This test provided an estimate of compliance (C), from the definition of C = ΔV/ΔP. The obtained values of C divided by the models’ initial volume (V_0_ = 9.38 ml) yielded the distensibility D = C/V_0_.

The repeatability of the method was ensured. For one model (0.7 mm vertical), five P measurements were taken for each dV and the standard deviation for each set of measurements was calculated, assessing the ability of obtaining the same value on repeated tests for the same input [21].

### Applicability to patient-specific modelling

Having identified a range of distensibility that can be implemented by varying the thickness of the material, two challenging cases were selected to assess the feasibility and the usefulness of the method.

In the first case, a 3-month-old patient with hypoplastic left heart syndrome (HLHS) and aortic coarctation (2.7 mm narrowing) was identified. The whole heart sequence was used to reconstruct the 3D anatomy of ascending, transverse and descending aorta using commercial software (Mimics, Materialise). Information on the change of aortic cross-sectional area (A) from the aortic flow CMR sequence was combined with P data from the catheterization procedure in order to estimate distensibility $D=\frac{1}{A_{\mathrm{dias}}}\frac{\mathrm{dA}}{\mathrm{dP}}$, where A_dias_ = cross-sectional area at end diastole. Having thus obtained an indication of patient-specific aortic distensibility, a model was manufactured extruding the appropriate thickness in order to implement the correct distensibility. Connectors for insertion of the model into an experimental rig were also included. This model was later mounted in a mock circulatory system [7] for hydrodynamic tests.

In the second case, a 33-year-old patient with severe pulmonary regurgitation and a dilated right ventricular outflow tract (RVOT) was selected. This patient was indicated as a suitable candidate for receiving a novel stent-graft for percutaneous pulmonary valve implantation (PPVI). The anatomy of the RVOT and pulmonary arteries bifurcation was derived from the CMR whole heart sequence and reconstructed in 3D (Mimics, Materialise). In order to test the patient’s suitability for insertion of the novel PPVI device, a compliant model was printed. This application shows the feasibility of such rapid prototyped compliant phantoms for device testing and patient selection. In this case, because of the lack of catheterisation pressure data, distensibility was not quantified, but a physiologically representative value was rather implemented.

## Results

### Quantification of distensibility in a simple anatomical model

**Figure 2:**
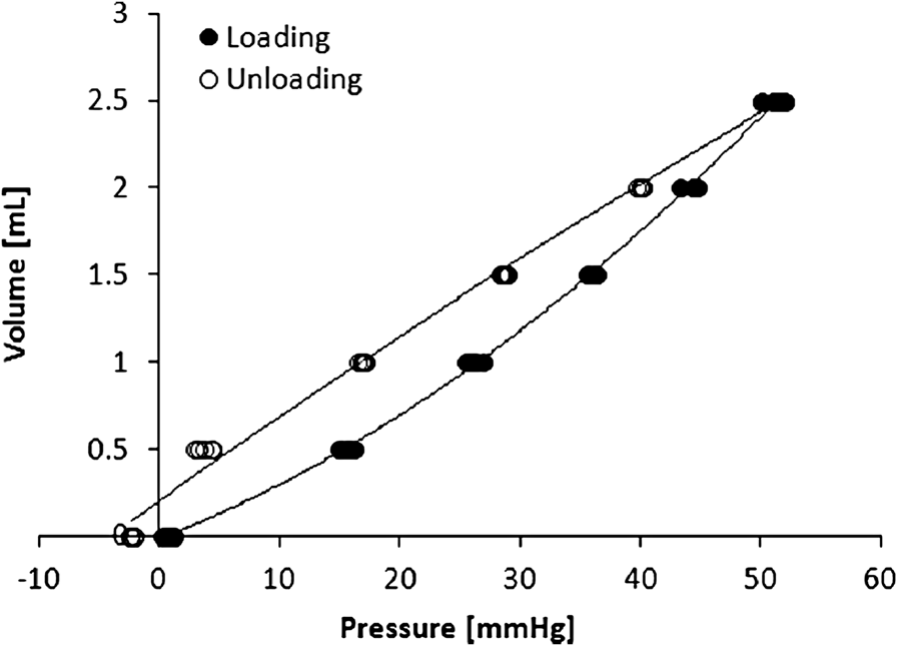
**Compliance testing of aortic samples. **Sample of the pressure-volume relationship resulting from a compliance test. The loop exhibits an area of hysteresis due to the viscoelastic behaviour of the material. Five data points, collected for the 0.7 mm thick “vertical” model, are shown at each dV, demonstrating the repeatability of the method.

**Figure 3:**
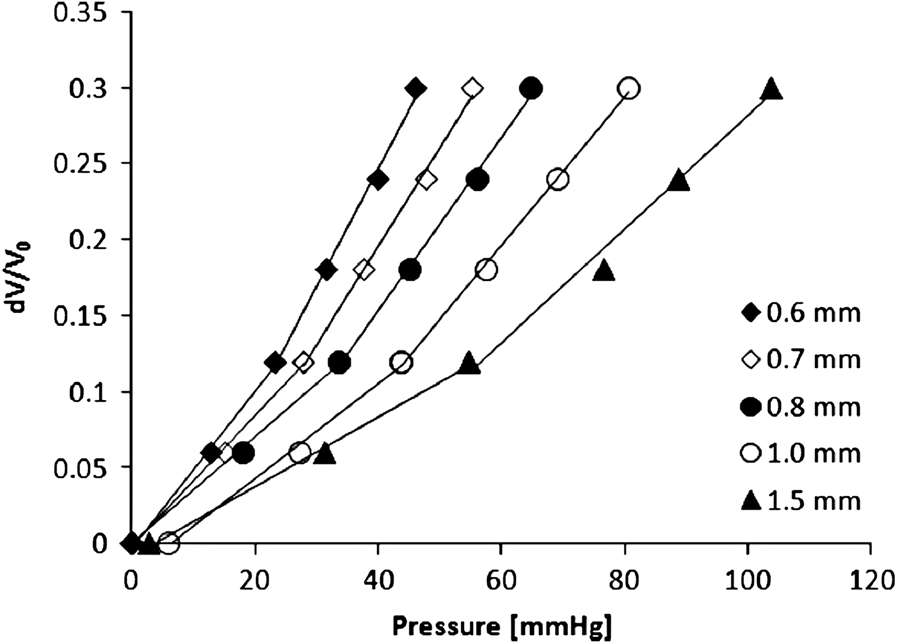
**Compliance changes with increasing wall thickness. **Change in pressure for corresponding changes in volume (dV) normalised for the initial volume of the sample (V_0_) for the range of tested thicknesses (0.6 – 1.5 mm).

**Figure 4:**
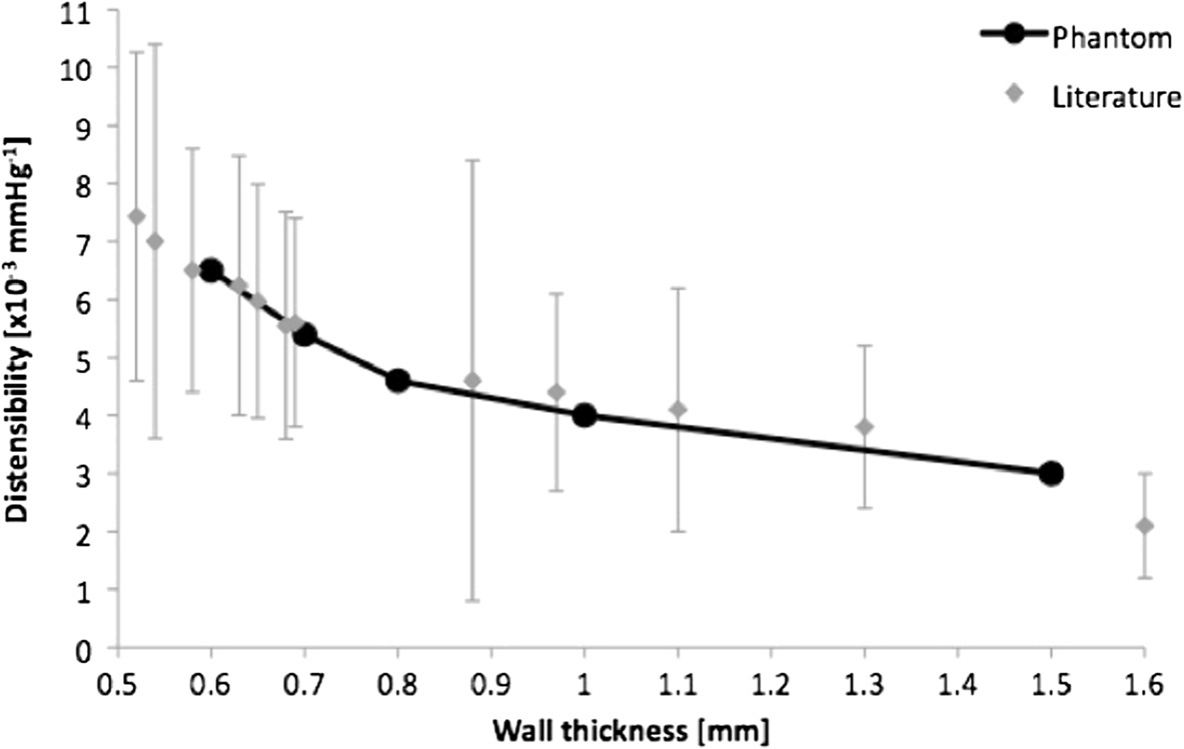
**Physiological distensibility range. **Relationship between increasing wall thickness and decreasing distensibility (D) for the compliant aortic models (*black circles*). The values of D implemented by the rapid prototyped models are within a physiological range, as shown by the comparison with a clinical range of D values (*grey diamonds*) for different arteries, including ascending and descending aorta [16,17], pulmonary artery [18] and carotid artery [19].

**Figure 5:**
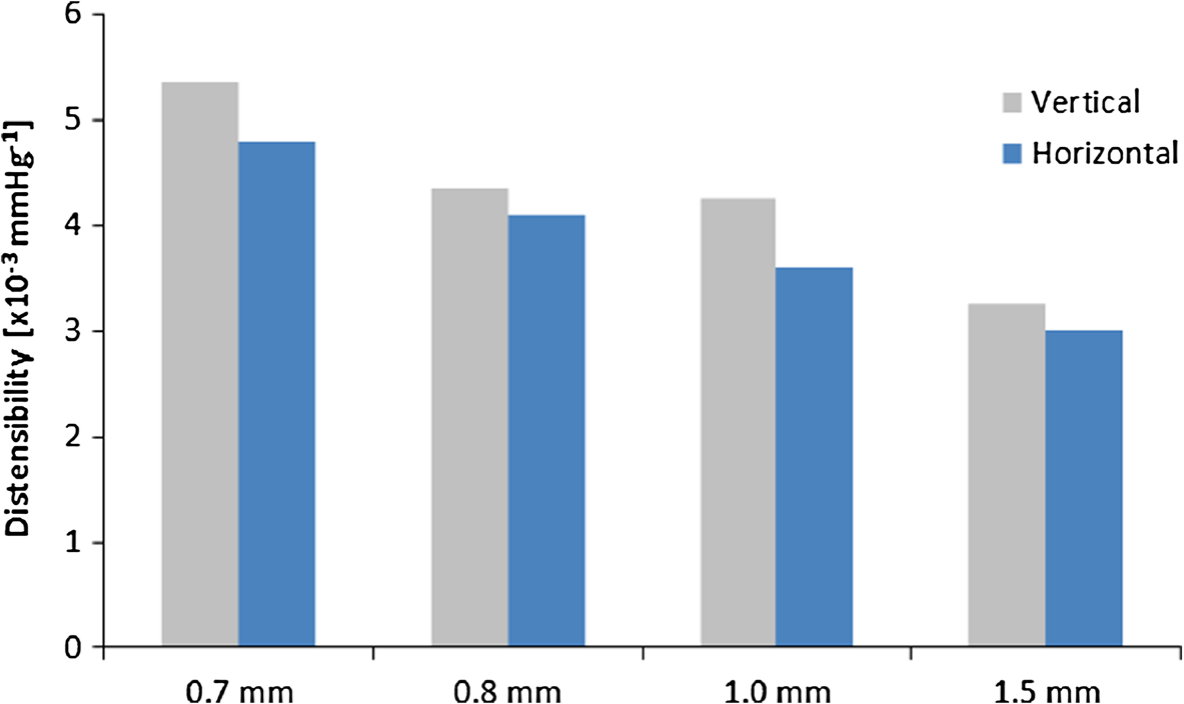
**Difference between printing modes. **The vertical mode for printing always resulted in stiffer models. Note: the 0.6 mm thickness case is not reported as the vertical specimen was damaged and the comparison could not be performed.

All descending aortic models were well representative of the original anatomy. Printing time was fast, demonstrating that the technique can generate a model within 12 hours. The result of the compliance tests highlighted the viscoelastic behaviour of the material, as indicated by an area of hysteresis in the P-ΔV relationship (an example of P-ΔV loop is provided in Figure 2). Measurements were highly repeatable, as shown by the small difference between multiple measurements (Figure 2) with standard deviations ranging between 0.2 and 0.7 mmHg. The relationship between P and dV/V_0_ highlighted the larger ΔP measured for the thicker models (Figure 3). This effectively translates into reduced distensibility, which was quantified from the slopes of the aforementioned relationship. The values of D for different thicknesses are shown in Figure 4 and were found to be in a physiological range, from 6.5 × 10^-3^ mmHg^-1^ at 0.6 mm thickness to 3.0 × 10^-3^ mmHg^-1^ at 1.5 mm thickness (Figure 4). Moreover, it was observed that the models that were printed in the vertical orientation were always more compliant than those printed horizontally (Figure 5), except for the 0.6 mm case for which the comparison is missing since one of the specimens (vertical) was damaged during the test. Vertical models were 11.0 ± 5.2% more distensible than horizontal models.

### Applicability to patient-specific modelling

**Figure 6:**
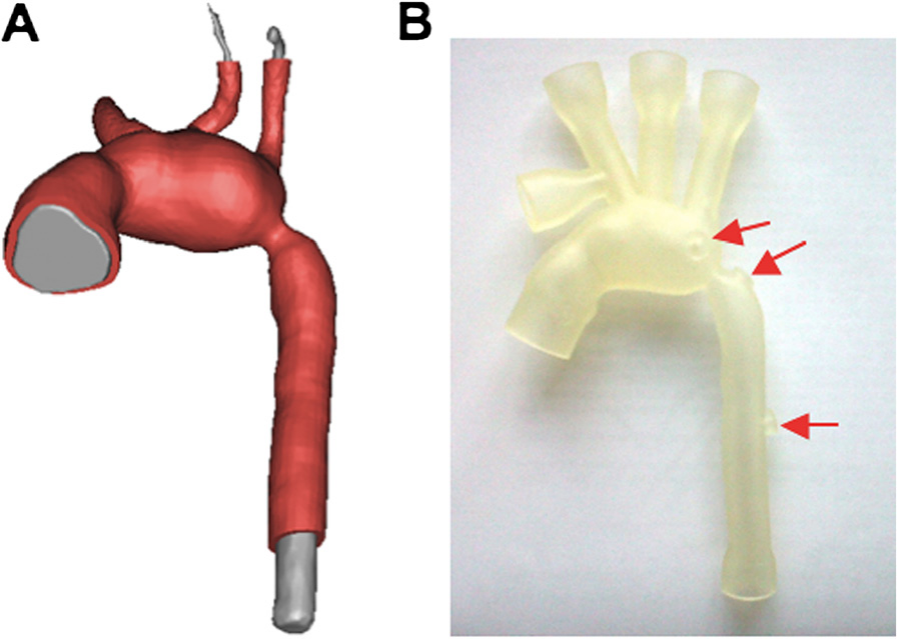
**Models for hydrodynamic tests, example of hypoplastic aortic arch. **Extruding a model of hypoplastic aorta with wall thickness appropriate for implementing patient-specific distensibility as derived from CMR data (**A**) and the finished model (**B**), including tapered connections for insertion in a mock circulatory system for hydrodynamic tests, and side ports for pressure measurements at different locations (the latter indicated by the red arrows).

Estimate of ascending aorta distensibility from CMR and catheterisation data in the HLHS patient yielded a value of 4.6 × 10^-3^ mmHg^-1^. Consequently, considering the relationship between wall thickness and distensibility (Figure 4), the HLHS 3D aortic model was extruded at 0.8 mm (Figure 6A). This phantom (Figure 6B) was successfully inserted in a mock circulatory loop, where it was able to withstand pressures in a physiological range for a patient with HLHS (70/40 mmHg).

**Figure 7:**
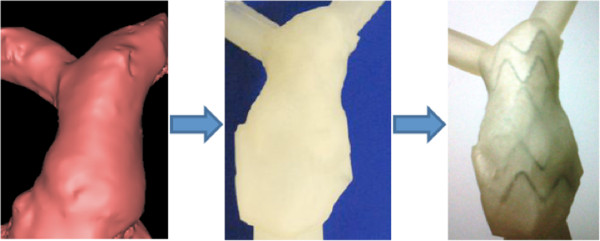
**Models for device testing, example of right ventricular outflow tract. **A patient-specific model of right ventricular outflow tract (RVOT): 3D volume derived from CMR data (*left*), from which a physical phantom is rapid prototyped (*centre*) and then used for physical insertion of a stent-graft (*right*) for assessing patient’s suitability for the device.

Rapid prototyping of RVOT model was also successful and well representative of the original anatomy, as shown in Figure 7. Once the model was manufactured, physical insertion of the novel stent-graft for PPVI was achieved and, in this case, indicated that the patient was suitable for device implantation [22,23]. The wall thickness of the RVOT model was 1.5 mm.

## Discussion

PolyJet technique has been described as anatomically accurate with excellent finishing in vascular applications, including aortic modelling [24,25], and other clinical applications, e.g. maxillofacial modelling [17]. These applications, however, involved rigid phantoms. In order to include the elastic nature of blood vessels, while taking advantage of the accuracy and speed of the technique, a compliant PolyJet-compatible material has been identified. This study quantifies the distensibility of said material (TangoPlus FullCure®) and highlights the relationship between wall thickness and its distensibility. From the obtained range of D values (Figure 4) we can infer in fact that TangoPlus FullCure® is a suitable material for modelling *invitro* the distensibility of arteries, including ascending and descending aorta [26,27], pulmonary artery [28] and carotid artery [29] as shown in Figure 4, based on values derived from the literature. On the other hand, this material appears to be an unsatisfactory choice for modelling the venous system, since the compliance of a systemic vein is about 24 times that of its corresponding systemic artery [30]. One possible alternative for generating venous phantoms may be represented by latex, due to its higher elasticity [31], but this material is currently not suitable for rapid prototyping.

PolyJet machines can also easily fabricate patient-specific arterial models in TangoPlus with non-uniform thickness for those cases in which a significant variation in wall elasticity is established in the region of interest. A good example for this application can be identified in the use of homograft patches to augment arterial lumens during surgical treatment of congenital heart diseases such as Tetralogy of Fallot, pulmonary stenosis and hypoplastic left heart syndrome. In the latter, where during the first stage of palliation the aortic arch is usually reconstructed using a homograft patch, it has been shown that D varies significantly between the reconstructed arch and the native descending aorta, the arch being stiffer [32]. Modelling of such a physiology for *invitro* purposes would certainly benefit from a distensible phantom with non-uniform wall thickness, which could replicate more closely the *invivo* situation. A physical model of a patient-specific hypoplastic arch, including aortic coarctation, with non-uniform wall thickness has been presented in this paper, demonstrating the implementation of patient-specific distensibility in the rapid prototyped phantom.

Qualitatively, good anatomical finishing was achieved not only in the RVOT model of an adult patient, but also in a small-sized model of HLHS with aortic coarctation, including connections for *in*-*vitro* tests. The latter can be easily added to the desired model and purposely designed to allow for insertion of catheters for measurements in specific locations, while also allowing for device implantation and testing such as in the RVOT case.

Producing such models in a fast and relatively inexpensive manner has multiple advantages:

1. More realistic *in*-*vitro* tests: mock circulatory loops could easily include compliant models with fine patient-specific anatomical and distensibility characteristics;

2. Device testing: the usefulness of rapid prototyped models for testing novel devices has been demonstrated in terms of assessment of patient’s suitability [20] and procedural planning [33], and deformable phantoms with realistic distensibility would further aid in this context;

3. Validation of computer models: *in*-*vitro* tests carried out in deformable phantoms with known and carefully defined wall properties can be a source of validation for fluid–structure interaction computational models, including 4D flow experiments in the CMR scanner and visualisation experiments with deformable phantoms for fluid dynamic considerations. Experiments involving compliant phantoms imaged with 4D flow are the object of on-going research and comparison of results obtained in these setups with *in-vivo* data is indeed an interesting area of future work.

### Limitations and methodological considerations

While a full data set is here presented for the models printed in the “horizontal” mode, the 0.6 mm “vertical” specimen was damaged during the experiment and thus this point is unavailable. From a qualitative point of view, it is noted that the thin-walled specimens, although easily withstanding a ΔP of about 46 or 55 mmHg (0.6 and 0.7 mm horizontal specimens), appeared to be prone to tearing. This should be taken into consideration when manufacturing a model, especially in the case of bifurcations and connections to tubing or other structures.

It was observed that calculating two slopes instead of one for the estimate of distensibility from the pressure-volume relationship provided a better fit. This was thus applied in the analysis of the data, as shown in Figure 3.

The major limitation of this study is the small number of samples, due to a limited number of available models. Considering this limitation, observations on printing orientation should be drawn with caution, although the data presented in Figure 5 suggest that this point should be taken into consideration when printing a model and could potentially be further explored in future work. On the other hand, despite this limitation, preliminary observations on distensibility have physiological relevance and the reliable nature of the measurements is supported by the results from the repeatability test.

At present, T1 and T2 times for the compliant compound described in this Technical Note are not available.

## Conclusion

TangoPlus FullCure® is a material which combines the advantages of PolyJet manufacturing, above all geometrical accuracy and speed of fabrication, with elastic properties that render it a suitable and convenient option for rapid prototyping arterial flow phantoms. However, the material is too rigid for being employed in simulating venous structures *in*-*vitro*. This paper also demonstrates the applicability of PolyJet technique for manufacturing realistic models of congenital heart disease, with very representative anatomical finishing.

## Competing interests

PV and RZ work at Materialise NV, Leuven, Belgium. There are no competing interests.

## Authors’ contributions

GB and SS were involved in the design of the study, creation of models from CMR data, acquisition and analysis of distensibility data, and drafting the manuscript; PV and RZ provided expertise in rapid prototyping and 3D modelling, and printed the aortic phantoms; AMT was involved in the design of the study, and drafting the manuscript and revising it critically for important intellectual content. All authors read and approved the final manuscript.
